# Marginal Indication for Thoracoscopic Surgery for Neonatal Bochdalek Hernia: “Anchor‐Shaped Closure” Technique for the Patient's Own Residual Diaphragm Using a Loop Needle Device

**DOI:** 10.1111/ases.70032

**Published:** 2025-02-16

**Authors:** Chihiro Kedoin, Koshiro Sugita, Toshio Harumatsu, Yumiko Tabata, Yumiko Iwamoto, Masato Ogata, Lynne Takada, Ayaka Nagano, Yudai Tsuruno, Masakazu Murakami, Keisuke Yano, Shun Onishi, Takafumi Kawano, Satoshi Ieiri

**Affiliations:** ^1^ Department of Pediatric Surgery, Research Field in Medicine and Health Sciences, Medical and Dental Sciences Area, Research and Education Assembly Kagoshima University Kagoshima Japan

**Keywords:** Bochdalek hernia, congenital diaphragmatic hernia, thoracoscopic repair

## Abstract

**Introduction:**

Surgical procedures to avoid using artificial materials require ongoing discussion. We herein report a case of thoracoscopic repair for congenital diaphragmatic hernia (CDH) via anchor‐shaped closure with the patient's own residual diaphragm using a loop needle device.

**Patient and Surgical Technique:**

A 2‐day‐old boy prenatally diagnosed with CDH underwent thoracoscopic repair after his respiratory and circulatory conditions had stabilized. The defect was a typical Bochdalek CDH, approximately 2.5 × 4 cm. The herniated organs of the thoracic cavity were the stomach, small intestine, colon, spleen, and left kidney. After these organs had been gently returned to the abdominal cavity under artificial pneumothorax, the medial side of the defect was closed in the anterior and posterior directions with six stitches of Loeder's knot using 3–0 non‐absorbable sutures. However, the lateral third of the defect was relatively large and difficult to close in the anterior and posterior directions. We therefore opted for closure by fixing the diaphragm to the chest wall and driving five external costal sutures using a loop needle device. The diaphragmatic defect was thus closed in an “anchor‐shaped” fashion using the patient's own residual diaphragm. This technique allows artificial membranes to be avoided in infants.

**Discussion:**

Considering the possibility of recurrence and complications, the indications for our procedure are limited; however, we believe that there are cases in which this procedure can provide a cure. Our proposed technique may be effective in closing relatively large diaphragmatic defects.

## 1 Introduction

Thoracoscopic repair for neonatal CDH has advantages in terms of improved cosmetic outcomes, shortened hospital stay, and an improved postoperative respiratory outcome [1, 2, 3, 4, 5]. Thoracoscopic repair also has the technical advantages of easily returning organs to the abdominal cavity using artificial pneumothorax, and it is easy to confirm the exact size of the diaphragmatic defect.

In the past, the recurrence rate of thoracoscopic surgery was higher than that of open surgery [4, 5], but with the accumulation of surgical experience, the surgical outcomes have improved to a level similar to that of the open approach in recent years [3, 6, 7, 8, 9]. However, the indications for thoracoscopic surgery in cases with large defects and long‐term outcomes remain controversial. Large defects sometimes require patch closure using artificial membranes. In recent years, it has been reported that thoracoscopic repair can be performed without increasing the recurrence rate in select cases [6]. Although ongoing research is exploring optimal patch materials, sizes, and shapes to minimize recurrence in thoracoscopic repair [1, 6], avoiding the use of artificial materials in pediatric surgery remains an important concern considering long‐term growth and unexpected infection.

We herein report a thoracoscopic repair for CDH via anchor‐shaped closure with the patient's own residual diaphragm using a loop needle device.

## 2 Materials and Surgical Technique

**FIGURE 1:**
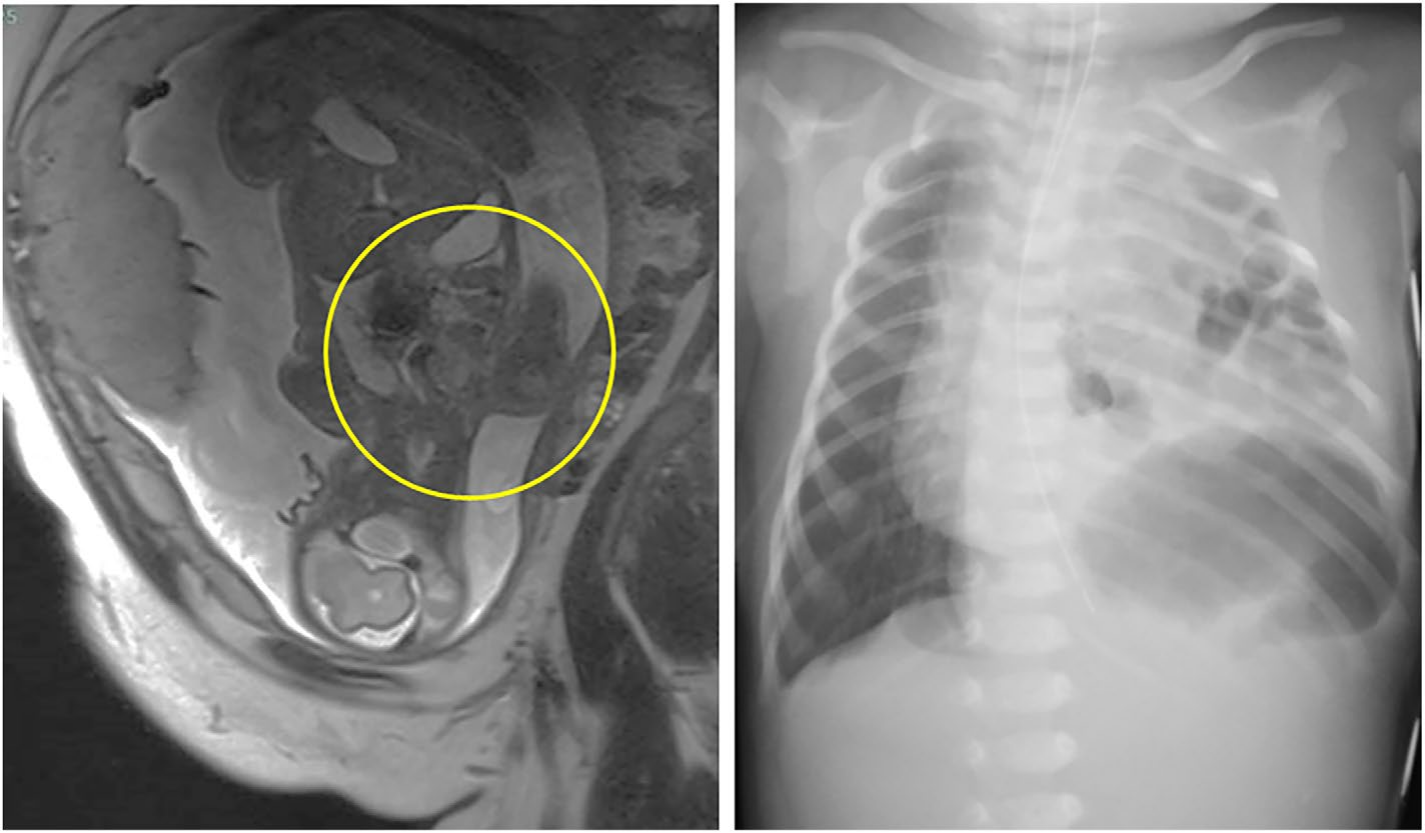
Image findings of fetal MRI and chest X‐ray at birth. Left: Fetal magnetic resonance imaging, right: Chest X‐ray at birth.

The patient was a male infant prenatally diagnosed with CDH at 22 weeks of gestation (Figure 1). He was delivered by Caesarean section at 39 weeks of gestation, weighing 3288 g. Immediately after birth, he was intubated and managed with conventional mechanical ventilation but did not require advanced respiratory support, such as nitric oxide, high‐frequency oscillatory ventilation, or extracorporeal membrane oxygenation (Figure 1). After 2 days of stabilization of the respiratory and circulatory conditions, thoracoscopic repair was performed. The patient was placed in the right lateral position, and a 5‐mm port as a camera port was inserted through the fifth intercostal space using optical methods. Subsequently, 5‐ and 3‐mm ports were inserted through the anterior and posterior axillary lines at the fifth and sixth intercostal spaces, respectively.

**FIGURE 2:**
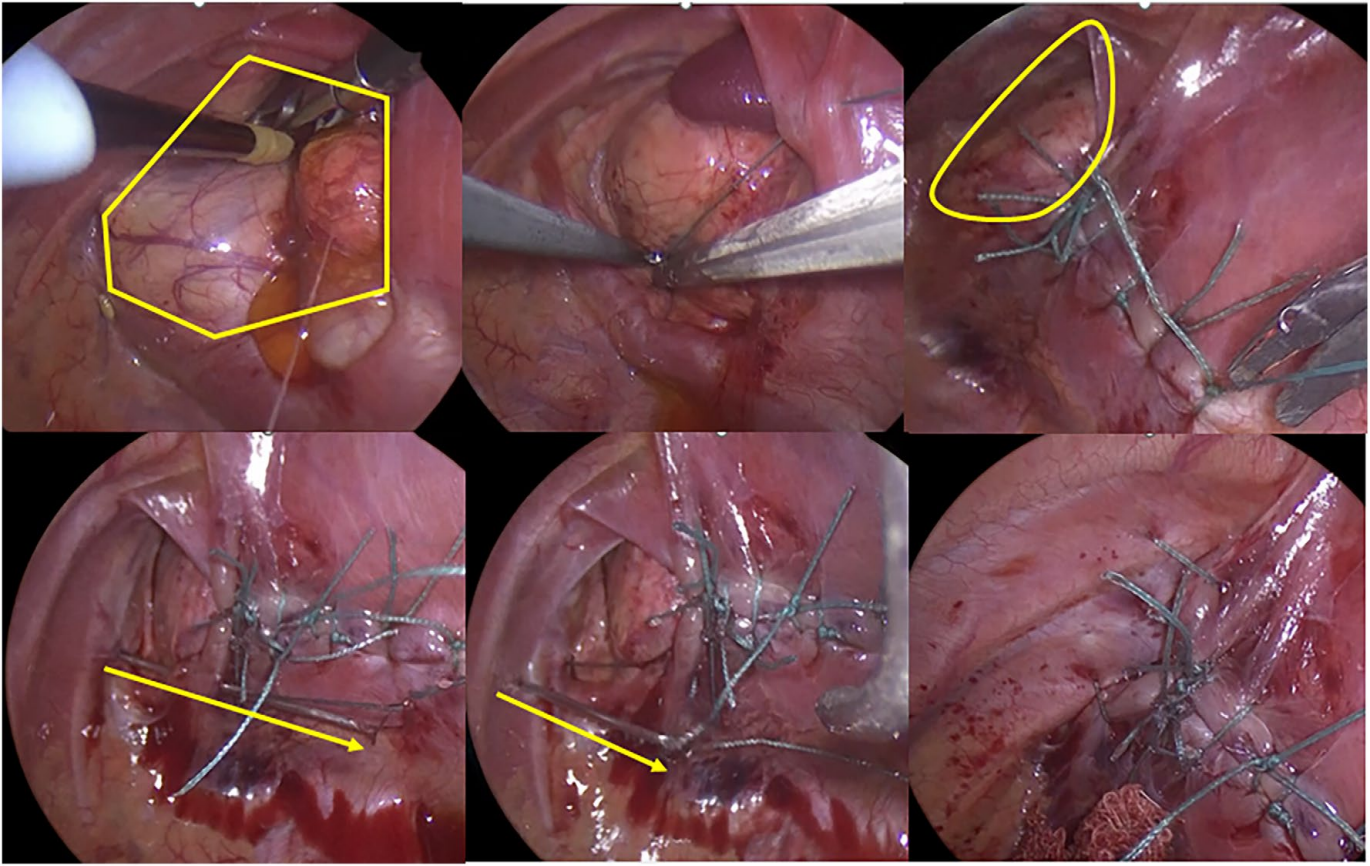
Intraoperative findings and surgical procedure. Upper panel: Left, yellow frame, defect; Center, first suture to the median of the hernia; Right, yellow frame, residual defect. Lower panel: Left, yellow arrow, insert a loop needle device below the ribs, and release the suture; Center, yellow arrow, insert a loop needle device above the ribs and retrieve the suture; Right, fix the diaphragm to the lateral thoracic wall.

**FIGURE 3:**
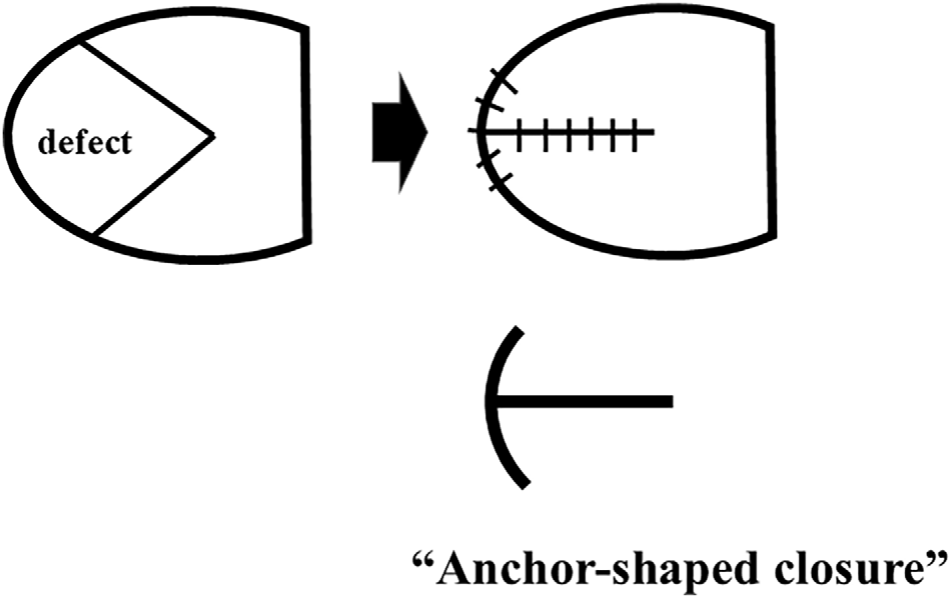
Schematic illustration of the “anchor‐shaped closure” technique.”

Intraoperative findings and surgical procedures are shown in Figure 2 and supporting video as Video S1, respectively. He had an isolated CDH, and the defect size was approximately 2.5 × 4 cm. It was classified as Defect B (< 50% defect of the diaphragm) according to the CDH Study Group (CDHSG) classification system. Based on this, the CDHSG staging system classified him as Stage II. The herniated organs in the thoracic cavity were the stomach, small intestine, colon, spleen, and left kidney. After gentle reduction of the herniated organs into the abdominal cavity under artificial pneumothorax, the median diaphragmatic limb was initially sutured using a 3–0 non‐absorbable thread (Echibond Excel) to prevent organ herniation. The medial side of the defect was then closed in the anterior and posterior directions with a total of six stitches. However, the lateral third of the defect is relatively large and difficult to close in the anterior and posterior directions. Thus, we decided to close the defect by fixing the diaphragm to the chest wall and driving five external costal sutures using a loop needle device. The loop needle device was inserted into the thoracic cavity through the intercostal space. The needle was inserted into the thoracic cavity through the lower rib. It was moved through the edge of the medial diaphragm from caudal to cranial and the suture was released. And then, the needle was inserted into the thoracic cavity through the upper rib and the suture was retrieved. After placing two sutures in the center of the medial diaphragm, the medial diaphragm was displaced in the plane by gently applying tension to both sutures simultaneously to reduce the risk of tearing the medial diaphragm. And then the sutures were tied one by one. One additional suture was placed in the center between the two threads, and one additional suture was placed on the outer sides of the two threads. Finally, the lateral side of the diaphragmatic defect was closed with the patient's diaphragm in an “anchor‐shaped” fashion using the patient's own residual diaphragm (Figure 3).

**FIGURE 4:**
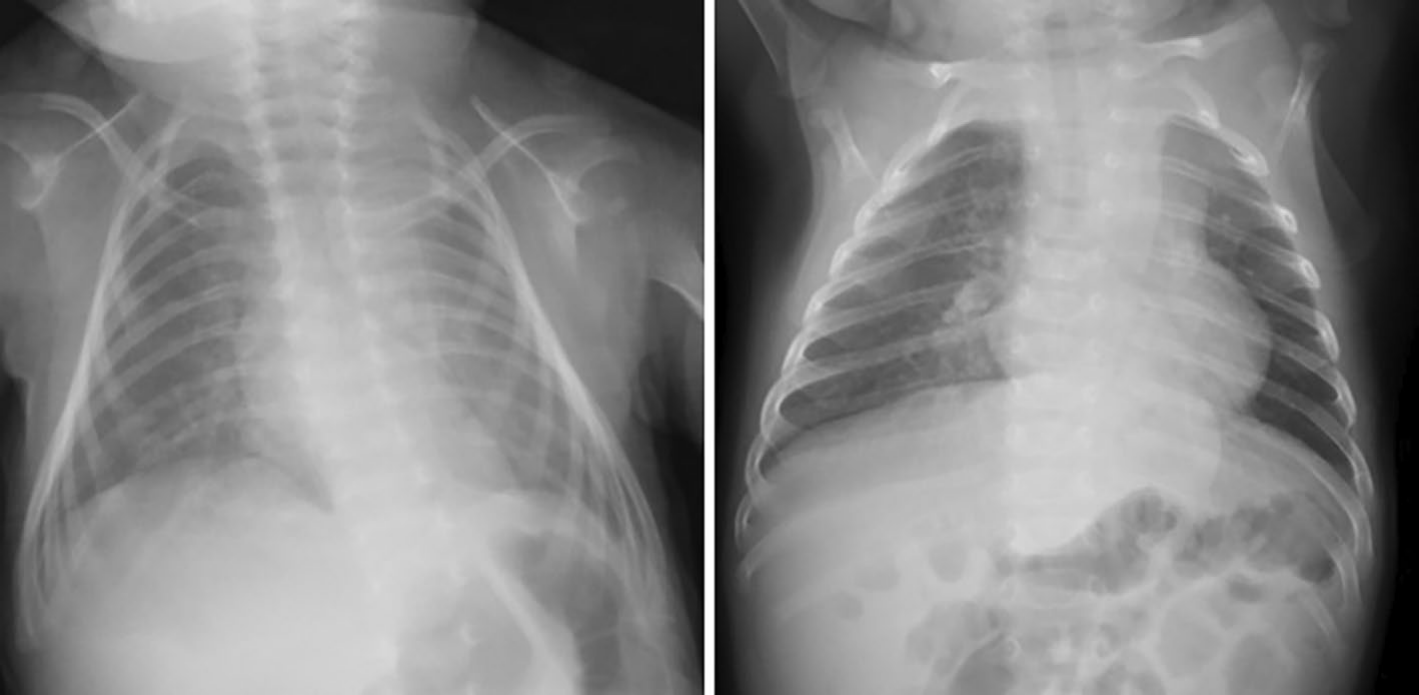
Postoperative X‐ray. Left: At the time of discharge, right: 2 months after surgery.

After surgery, the ventilator (FiO_2_ 0.3) was slowly weaned, and he was extubated on post operative day 5. After surgery, pleural effusion occurred due to lung expansion, and chest drainage was required. However, even after extubation, oxygen demand remained stable with CPAP ventilation alone. He was discharged on post operative day 27 when oral intake without respiratory effort was established. Postoperative X‐rays taken at the time of discharge and 2 months after surgery (the last one) are shown in Figure 4. He has been asymptomatic at present, 8 months after surgery.

## 3 Discussion

CDH is classified according to the size of the defect, as determined by the CDH Research Group [10]. For types C and D, which are large defects, an artificial membrane must be used, regardless of the surgical approach [1]. Type A, which involves small defects, is the most appropriate indication for thoracoscopic surgery. In cases such as type B, where there is little tissue on the posterior lateral side of the diaphragm, if an attempt is made to close the defect directly by suturing, the suture tension becomes too strong and recurrence is likely to occur, so an artificial membrane is sometimes used even in children [6]. While repair of CDH is essentially a straightforward procedure involving organ reduction and closure of hernia defects, similar to other hernia repairs, postoperative recurrence remains a concern. One reason for recurrence is hypoplasia or defect of the lateral side of the patient's diaphragm, which is a critical portion of the closure of CDH. The current case is compatible with type B, and our technique resolved this problem by approximating the medial patient's diaphragm in a mediolateral direction to close the lateral side of the defect. Kamran A et al. reported that the CDH with type B defect was thoracoscopically repaired partially (11/21, 52.4%) or entirely (10/21, 47.6%) with a patch. Our technique appears to be applicable to patients with type B CDH who have undergone partial repair with a patch [11]. In particular, those closer to type A would be ideal candidates. A loop needle device penetrates both the superior and inferior ribs using in our technique. If it is possible to pass the needle up and down within the intercostal space, it may not be necessary to pass the needle above and below the rib. According to a report of using the needle device to fix patches in thoracoscopic surgery, both of the intracostal manipulation and the manipulation passing above and below the rib are used [6]. There is a high risk of damaging the intercostal arteries and nerves due to the narrow intercostal space in neonates. We think that intracostal manipulation should be done with caution in neonate. Our technique is different from intrathoracic suturing in terms of simply technical ease and tension during tying. The diaphragmatic muscle in newborns is more fragile and prone to tearing, and the limited surgical space makes it difficult to fully mobilize the diaphragm and securely repair the defect [11]. In particular, tension is difficult to disperse among several sutures when tying knots thoracoscopically, and an excessive amount may be placed on each suture during the repair [12]. We perform the surgery in neonatal period, and this technique ligating subcutaneously may be more effective in preventing recurrence than intrathoracic manipulation. A potential risk with our technique is increased tension in the lateral direction on the neo‐diaphragm toward the chest wall. Our next step is to define the marginal indication for “Anchor‐shaped Closure.”

“Anchor‐shaped closure” may be a potential candidate technique to ensure the closure of the posterolateral diaphragm without using an artificial membrane. Avoiding artificial materials is the most ideal treatment for patients, and we preserve a patch closure as a second‐stage surgical option. Our patient was able to be discharged with his own diaphragm. Our treatment strategy for selective patients with CDH with defect B may provide two options at the time of recurrence: direct closure using the patient's own diaphragm again or patch closure could be performed. As the long‐term outcomes of this surgical procedure are unknown, it is necessary to continue accumulating cases and clarify the long‐term results.

## Author Contributions

C.K. and K.S. drafted the manuscript, and K.S. and S.I. supervised the writing of the manuscript. T.H., Y.T., Y.T., Y.I., M.O., L.T., M.M., K.Y., S.O., T.H., and T.K. performed perioperative management. All authors have read and approved the final manuscript.

## Disclosure

The authors have nothing to report.

## Conflicts of Interest

The authors declare no conflicts of interest.

## Supporting information


**Video S1.** Demonstration of thoracoscopic repair of the diaphragmatic hernia using the “anchor‐shaped closure” technique.

## Data Availability

The data that support the findings of this study are available on request from the corresponding author. The data are not publicly available due to privacy or ethical restrictions.
